# Retained Products of Conception: An Atypical Presentation Diagnosed Immediately with Bedside Emergency Ultrasound

**DOI:** 10.1155/2016/9124967

**Published:** 2016-02-07

**Authors:** Kristin Adkins, Joseph Minardi, Erin Setzer, Debra Williams

**Affiliations:** ^1^West Virginia University School of Medicine, Morgantown, WV, USA; ^2^Department of Emergency Medicine, West Virginia University, 7413B HSS, 1 Medical Center Drive, Morgantown, WV 26506, USA

## Abstract

*Background*. Retained products of conception is an important diagnosis to consider in patients presenting with postpartum complaints. Bedside ultrasound is a rapid, accurate, noninvasive modality to evaluate these patients.* Objective*. To report an atypical case of retained products of conception diagnosed with bedside ultrasound in the emergency department.* Case Report*. A 27-year-old female who was 1-month postpartum presented with vaginal bleeding, pelvic pain, and no fever. At the time of initial H&P, bedside ultrasound revealed echogenic material within the endometrial cavity with blood flow seen by color Doppler consistent with retained products of conception. The bedside ultrasound rapidly narrowed the differential and allowed a definitive diagnosis immediately. Ob/Gyn was consulted and dilation and curettage was performed in the operating room.* Conclusions*. Retained products of conception is an important diagnosis for the emergency physician to consider in at-risk patients. The sonographic findings are easily obtained and interpreted by emergency physicians. Earlier diagnosis of this disease process should lead to more focused patient evaluations and management.

## 1. Introduction

Postpartum complaints are common in the emergency department. Retained products of conception (RPOC) is one of the most important differential considerations in these patients. RPOC should be suspected if a postpartum patient presents with symptoms of endometritis or hemorrhage, specifically pelvic pain, vaginal discharge or bleeding, and possibly fever [1]. The presentation can be variable. Traditionally, ultrasound is ordered to evaluate these complaints, but radiology ultrasound is frequently only available during limited hours. The common application of bedside ultrasound by emergency physicians should translate well to evaluating this diagnosis. The typical sonographic findings are relatively easy to identify. Emergency physicians should be capable of recognizing these findings leading to more rapid diagnosis, decreased use of other resources, and more expeditious management. Although this diagnosis is commonly made by obstetrician/gynecologists and radiologists, to the authors' knowledge, there have been no descriptions of this diagnosis and its findings by emergency physicians using bedside ultrasound.

## 2. Case Report

A 27-year-old female presented to the emergency department with excessive vaginal bleeding and an episode of syncope. She was approximately one-month postpartum from a vaginal delivery. She reported heavy bleeding during delivery but had been asymptomatic until three days prior to presentation when she noticed some vaginal spotting. Approximately 30 minutes prior to presentation, she experienced a syncopal episode and reported heavy vaginal bleeding associated with mild suprapubic pain. She denied other symptoms. Her past medical and surgical histories were otherwise unremarkable.

Initial vital signs were BP 118/70, pulse 103, respirations 18, temp. 36.6, and pulse ox 100%. On physical examination, she appeared anxious and pale with small abrasion over the bridge of her nose. Her abdomen was tender to deep palpation over the pubic area, and her uterus was not palpable. Pelvic exam revealed blood and clots in the vagina, initially occluding the view of the cervix. The blood returned after suctioning. Cervical os was closed. The remainder of her physical examination was unremarkable.

Differential diagnoses included retained products of conception, uterine rupture, ectopic pregnancy, spontaneous abortion, and dysfunctional uterine bleeding.

**Figure 1:**
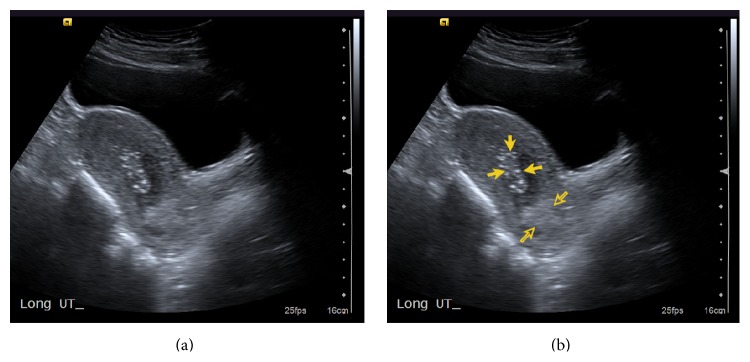
(a)* TA Sag Uterus*. Transabdominal sagittal view of the uterus shows thickened, heterogeneous, and hyperechoic material within the endometrial cavity. There is also echogenic material located in the lower uterine segment. (b) Opaque arrows indicate heterogeneous echogenic endometrial tissue. Open arrows indicate echogenic material in the lower uterine segment near the cervical os.

**Figure 2:**
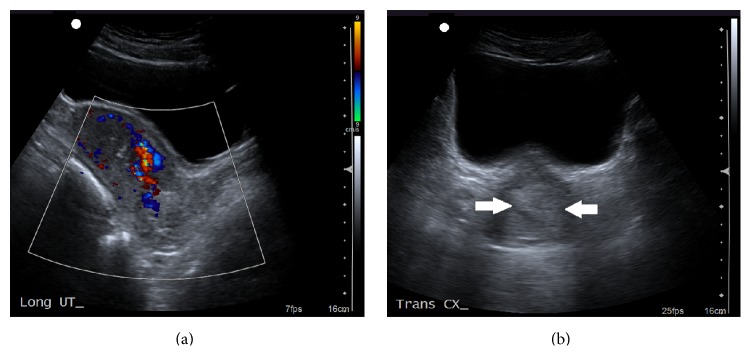
(a)* TA Sag Uterus, Color*. Transabdominal uterus showing blood flow within the echogenic intraendometrial tissue, making retained products highly likely. (b)* TA Trans Uterus*. Transabdominal transverse view of the uterus showing echogenic material within the lower endometrial cavity (arrows).

Bedside transabdominal ultrasound was performed by the emergency physician revealing heterogeneous and echogenic material within the endometrial cavity (as seen in Figure 1). Internal blood flow was demonstrated by color Doppler (as seen in Figure 2). The adnexa were unremarkable and there was no significant intraperitoneal free fluid. These findings were felt consistent with retained products of conception. Serum human chorionic gonadotropin level was negative and hemoglobin returned normal.

Ob/Gyn was consulted and the patient was taken for dilation and curettage, where placental tissue was removed from the cervical os and the uterus. Pathology confirmed that the tissue was chorionic villi and decidua. The patient tolerated the procedure well and had an unremarkable postoperative course.

## 3. Discussion

Retained products of conception is one of the most common reasons for readmission postpartum [2]. RPOC may be found after 1% of term pregnancies [3]. In one study, the median period from delivery to presentation was 11 days [2]. The diagnosis of RPOC should be considered in postpartum women who present with vaginal bleeding, fever, foul-smelling discharge, and abdominal or pelvic pain. This presentation is nonspecific, and clinical diagnosis alone has a high false-positive rate, up to 40% [4]. Dilation and curettage, the treatment of choice for RPOC, carries a risk of serious complications including uterine bleeding, perforation, infection, adhesions, and infertility. Because of this, it is important to rule out other diagnoses such as ectopic pregnancy, uterine rupture, or hematometra, to avoid the complications of unnecessary D&C. Ultrasonography is the diagnostic test of choice for evaluation of febrile postpartum patients due to its safety, accessibility, and cost benefits. However, the availability of radiology can be limited in emergency departments and the common use of bedside ultrasound by emergency physicians should translate well to this application. Typical sonographic findings include hyperechoic, intrauterine material with internal vascularity observed with color Doppler and high-velocity, low-resistance flow by spectral Doppler. Blood products may have a similar echogenic appearance but no internal flow [5]. According to Kamaya et al., the presence of any vascularity has a 96% positive predictive value for RPOC [6]. If RPOC is suspected, uterine evacuation may be necessary. This can be accomplished with sharp or suction curettage. If the bedside US exam is equivocal, antibiotics and close monitoring may be adequate. If there is an excellent response to antibiotics in the first 24 hours, surgical intervention may be avoided [7].

In summary, we report a case of retained products of conception, presenting atypically, rapidly and accurately diagnosed at the bedside by an emergency physician using clinical ultrasound. The clinical and sonographic findings are ones that all emergency physicians should recognize. Clinicians with a baseline skill set in clinical ultrasound can readily recognize the findings of this diagnosis.

## 4. Why an Emergency Physician Should Know about This

Retained POC is one of the more important diagnoses to be considered in the symptomatic postpartum ED patient. The typical sonographic findings are relatively straightforward and can be recognized by emergency physicians with existing skills in bedside ultrasound of the female pelvis. Making this diagnosis at the bedside may expedite management and limit the need for other investigations and consultations, particularly when radiology ultrasound is not immediately available.

## References

[1] Durfee S. M., Frates M. C., Luong A., Benson C. B. (2005). The sonographic and color Doppler features of retained products of conception. *Journal of Ultrasound in Medicine*.

[2] Matijevic R., Knezevic M., Grgic O., Zlodi-Hrsak L. (2009). Diagnostic accuracy of sonographic and clinical parameters in the prediction of retained products of conception. *Journal of Ultrasound in Medicine*.

[3] Wolman I., Altman E., Faith G. (2009). Combined clinical and ultrasonographic work-up for the diagnosis of retained products of conception. *Fertility and Sterility*.

[4] Sadan O., Golan A., Girtler O. (2004). Role of sonography in the diagnosis of retained products of conception. *Journal of Ultrasound in Medicine*.

[5] Laifer-Narin S. L., Kwak E., Kim H., Hecht E. M., Newhouse J. H. (2014). Multimodality imaging of the postpartum or posttermination uterus: evaluation using ultrasound, computed tomography, and magnetic resonance imaging. *Current Problems in Diagnostic Radiology*.

[6] Kamaya A., Petrovitch I., Chen B., Frederick C. E., Jeffrey R. B. (2009). Retained products of conception: spectrum of color Doppler findings. *Journal of Ultrasound in Medicine*.

[7] Carusi D. A. (2015). *Retained Products of Conception*.

